# Exploring Dynamics of Molybdate in Living Animal Cells by a Genetically Encoded FRET Nanosensor

**DOI:** 10.1371/journal.pone.0058175

**Published:** 2013-03-05

**Authors:** Yoichi Nakanishi, Syuntaro Iida, Hanayo Ueoka-Nakanishi, Tomoaki Niimi, Rie Tomioka, Masayoshi Maeshima

**Affiliations:** 1 Department of Biological Mechanisms and Functions, Graduate School of Bioagricultural Sciences, Nagoya University, Nagoya, Japan; 2 Multidisciplinary Pain Center, Aichi Medical University, Nagakute, Japan; 3 Division of Physics, Graduate School of Science, Nagoya University, Nogoya, Japan; 4 Department of Bioengineering Sciences, Graduate School of Bioagricultural Sciences, Nagoya University, Nagoya, Japan; 5 Department of Bioengineering Sciences, Graduate School of Bioagricultural Sciences, Nagoya University, Nagoya, Japan; University of Nottingham, United Kingdom

## Abstract

Molybdenum (Mo) is an essential trace element for almost all living organisms including animals. Mo is used as a catalytic center of molybdo-enzymes for oxidation/reduction reactions of carbon, nitrogen, and sulfur metabolism. Whilst living cells are known to import inorganic molybdate oxyanion from the surrounding environment, the *in vivo* dynamics of cytosolic molybdate remain poorly understood as no appropriate indicator is available for this trace anion. We here describe a genetically encoded Förester-resonance-energy-transfer (FRET)-based nanosensor composed of CFP, YFP and the bacterial molybdate-sensor protein ModE. The nanosensor MolyProbe containing an optimized peptide-linker responded to nanomolar-range molybdate selectively, and increased YFP:CFP fluorescence intensity ratio by up to 109%. By introduction of the nanosensor, we have been able to successfully demonstrate the real-time dynamics of molybdate in living animal cells. Furthermore, time course analyses of the dynamics suggest that novel oxalate-sensitive- and sulfate-resistant- transporter(s) uptake molybdate in a model culture cell.

## Introduction

Molybdenum (Mo) is an essential microelement for nearly all living organisms, in both prokaryotes and eukaryotes [1], [2]. It is utilized as a catalytic center in molybdoenzymes to oxidize and reduce carbon, nitrogen and sulfur metabolites, since this element is one of the transition metal elements with an oxidation state varying from +2 to +6. Most molybdoenzymes, such as xanthine oxidase, sulfite oxidase, aldehyde oxidase, nitrate reductase, and mitochondrial-amidoxime-reducing-component, contain a pterin-based Mo cofactor (Moco) [3], [4], while a bacterial nitrogenase includes iron-Mo cofactor (FeMo-co). The primary source of Mo for both cofactors is a molybdate oxyanion (MoO_4_^2−^), a major chemical form of Mo found in the natural water environment [1]. Organisms incorporate molybdate into cells by way of specific molybdate transporters or non-specific anion transporters in order to synthesize the cofactors described above [2], [5]. Meanwhile, uptake of too much Mo is toxic to organisms [1], [6]. In bacteria, the intracellular concentration of molybdate is maintained at the appropriate level by controlling molybdate transport in response to the intracellular molybdate concentration, as excessive molybdate is toxic [5]. In eukaryotic cells however, the dynamics of cytosolic molybdate and its related uptake systems are as yet unknown.

A specific, real time detection system is required in order to study molybdate dynamics in the intact eukaryotic cell, which has not been possible to date. A few techniques such as atomic emission spectrometry, inductively coupled plasma mass spectrometry (ICP-MS), and neutron activation analysis have been used to quantify the total molybdenum (not molybdate) in homogenized biological samples [7]. Radio tracing of ^99^Mo with a short half-life (66 h) has also been used to investigate the incorporation of Mo into organisms [8]. These methods do however present technical limitations for the identification of molybdate from total molybdenum. Moreover, their time- and spatial-resolution are unsuitable for analysis of the cellular and sub-cellular dynamics of molybdate in living eukaryotic cells.

Fluorescent-probes are widely used to investigate dynamics of intracellular ions and metabolites in living cells in combination with fluorescent microscopes. Genetically-encoded Förester-resonance-energy-transfer (FRET) nanosensors were recently developed by combining a pair of fluorescent proteins with a native ligand-binding protein [9], leading to the successful visualization of cellular calcium ion, sugars, amino acids, adenosine 5′-triphosphate (ATP), other small-molecule metabolites [10]–[15]. Genetically-encoded FRET nanosensors provide specificity and sensitivity by embedding a biochemical mechanism of ligand recognition with a conformational change of the ligand-binding protein.

A number of bacterial molybdate binding proteins have been biochemically characterized and their high-resolution crystal structures obtained. These include ModA, a periplasmic molybdate-binding-subunit of molybdate transporter ModABC [16]; Mop/ModG, a small cytoplasmic molybdate-storage-protein [17]; and ModE, a transcriptional regulator of a number of Mo-metabolism related gene operons, among other functions [18]–[20]. Among them, ModE is an authentic molybdate sensor in bacteria, and binds intracellular molybdate with a sub-micro molar affinity [21], changes its conformation by homo-dimerization in a molybdate dependent fashion, modulating its affinity to target DNA [22]. Here we report a ModE-based genetically coded nanosensor that transduces nanomolar concentrations of molybdate to a hetero FRET signal of CFP and YFP. We show the first example of real-time dynamics of trace molybdate in living animal cells. Furthermore, we propose a novel type of molybdate transport in a model culture cell.

## Materials and Methods

### Chemicals

Na_2_WO_4_ and Na_2_CrO_4_ were from Kanto Chemical co. Na_2_MoO_4_, K_2_SeO_4_, K2SO_4_ and other chemical reagents were from Wako Pure Chemicals.

### Genetic Construction of MolyProbe

Genes for CFP (CeruleanΔ11), two molybdate binding domains (MoBD) and YFP (cp157-Venus) were linked by linker DNA encoding optimized polypeptide connectors [23], [24]. To suppress spontaneous homologous recombination of MolyProbe gene in host cells, all parts were amplified from different sources by PCR. The gene for CeruleanΔ11, of which the c-terminal 11 aa were deleted, was derived from pECFP (Clontech) optimized for mammalian cell expression. The gene for cp157-Venus was generated by circular permutation from yEVenus of pKT90 (EUROSCARF) optimized for yeast expression. One MoBD was amplified from *E.coli* genomic DNA by PCR, another was originally designed by Gene-Synthesizer software [25], [26] and artificially generated from synthetic oligo-DNAs by ligase-chain reaction. Both MoBDs correspond to c-terminal halves of *E.coli* ModE. The DNA sequences of linkers between two MoBDs were selected from an original library of random-sized DNA fragment by expression screening. MolyProbe was assembled from these parts by multiple ligation, cloned into derivative of pBluescript II SK (+), and designated pYN627 for *E.coli* expression. For mammalian cell expression, the MolyProbe gene was subcloned into pcDNA3.1 (Invitrogen) and designated pYN723.

### Preparation of Recombinant MolyProbe

MolyProbe was expressed in *E.coli* DH5alpha transformed with pYN627 under a *lac* promoter. Protein expression was induced by overgrowth cultivation at 30°C for 30 h in LB medium without inducers. Recombinant protein was extracted by B-PER II extraction reagent (PIERCE), and separated by four-step column chromatography, anion exchange chromatography (Toyoperl-QAE-550C, Toso); hydrophobic chromatography (Toyoperl-Butyl-650M, Toso); anion exchange chromatography (UNO-Q, Bio-Rad) after dialysis; size exclusion chromatography (Superdex-200, GE Healthcare). The concentration of the purified protein was calculated from absorbance of 280 nm and a specific coefficient (8.75×10^4^ cm^−1^ M^−1^).

### Characterization of MolyProbe in vitro

Fluorescence of purified MolyProbe proteins (20 nM) was assayed in buffer A containing 20 mM Mops-Tris (pH7.2), 100 mM K-acetate, 2 mM MgCl_2_, 1 mM DTT and 0.025% (w/v) Tween-20 at 25°C. MolyProbe was excited at 430 nm (λ_max_ for CFP), and emission spectrum, 450 nm- 550 nm, was measured by fluorospectrometer RF-5300PC (Shimazu). The emission-intensity ratio (R_F530:F475_) was calculated from the fluorescent intensities of CFP (475±5 nm) and YFP (530±5 nm) with an original software. Data were fitted to Hill equation curve with KaleidaGraph software (Synergy software).

### Cell Culture and Transfection

HEK-293T cells were maintained in DMEM (Wako Pure chemicals) supplemented with 10% FBS at 37°C with 5% CO_2_. The cells were transfected with pYN723 using FuGENE6 transfection reagent (Roche), cultured in DMEM/F-12 medium without phenol red (Gibco) supplemented with 10% FBS, and then subjected to fluorescence analyses. About 60% of the cells exhibited fluorescence in the field of fluorescence microscopy. Concentrations of molybdate in the medium supplemented with 10% FBS were quite low (<10 nM).

### Fluorescence Analysis of Bulk Mammalian Cells

The cells transfected with MolyProbe were washed twice with ice cold PBS, dispersed with 0.25% trypsin, washed, and re-suspended in dye-free HBSS containing 5 mM Hepes-NaOH pH 7.4. The suspension were incubated with molybdate (1 mM) at 37°C or at 22°C, and the fluorescence measured by RF-5300PC.

### Live Cell Imaging

HEK-293T cells transfected with MolyProbe were grown on a glass-based dish (Asahi techno glass) for 2 days and subjected to real-time imaging. The culture medium was exchanged into dye-free HBSS containing 5 mM Hepes-NaOH pH 7.4 prior to assay. Molybdate was added to the medium just after the first laser scan. Fluorescence images were obtained using an inverted laser scanning confocal microscope (Olympus FluoView FV1000-D) with a 1.40 N.A., ×100 oil-immersion objective. MolyProbe was excited every 15 sec by a 440 nm LD laser, and fluorescence was separated to two channels by dichroic-mirrors and diffraction gratings (475±10 nm for CFP and 535±10 nm for YFP). A diffraction image of whole cells was obtained by using a 559 nm LD laser. Fluorescence images were processed using FluoView software (Olympus).

### Time-course Assay of Molybdate Uptake

HEK-293T cells transfected with MolyProbe was cultured for 3 days in a 96-well black plate (Asahi techno glass). Fluorescence data were obtained by Fluoroskan Ascent microplate fluorometer (Labsystems) with a set of band-pass filters (430±5 nm for excitation, 480±5 nm and 530±5 nm for emission, Asahi Spectra). The culture plate was pre-scanned and incubated for 30 min prior to the time-course assay. After zero-time data were scanned, molybdate was added to the medium, and then time point data were obtained sequentially. The plate was incubated at 37°C with 5% CO_2_ during the assay without scan times. Background fluorescence data were obtained by parallel culture of the cells transfected with mock vector (pcDNA3.1), used for calculation of the R_F530:F480_.

### Over-expression and Knockdown of *Hs*MoT2/MFSD5

*Hs*MoT2/MFSD5 cDNA was amplified by RT-PCR from total RNA of HEK-293T, cloned into a derivative of the pCMV-Script vector (Stratagene) and designated pYN769. Co-transfection of cells with pYN723 and pYN769 were performed by the polyethyleneimine transfection method using 25 kD linear- polyethyleneimine (Polyscience) [27], [28]. 46 ng pYN723, 9 ng pYN769 and 138 ng polyethyleneimine were mixed to make complex, dropped into pre-culture of the cell in 96-well black plates. For knockdown of *Hs*MoT2/MFSD5, a synthetic dsRNA MFSD5.779 (prepared from a pair of RNAs 5′-CCAUACAAGCUCUAUUUGAtt-3′ and 5′-UCAAAUAGAGCUUGUAUGGtt-3′, GeneDesign, Osaka) were co-transfected with pYN723 by X-tremeGENE siRNA reagent (Roche). Control experiments for both co- transfection was performed by replacement of effecter plasmid DNA or dsRNA by the same amount of mock DNA (pUC119). The level of *Hs*MoT2/MFSD5 mRNA was monitored using a real-time qPCR method, quantified by comparing with standard amounts of pYN769.

## Results

### Development of Molybdate Sensing Protein

**Figure 1:**
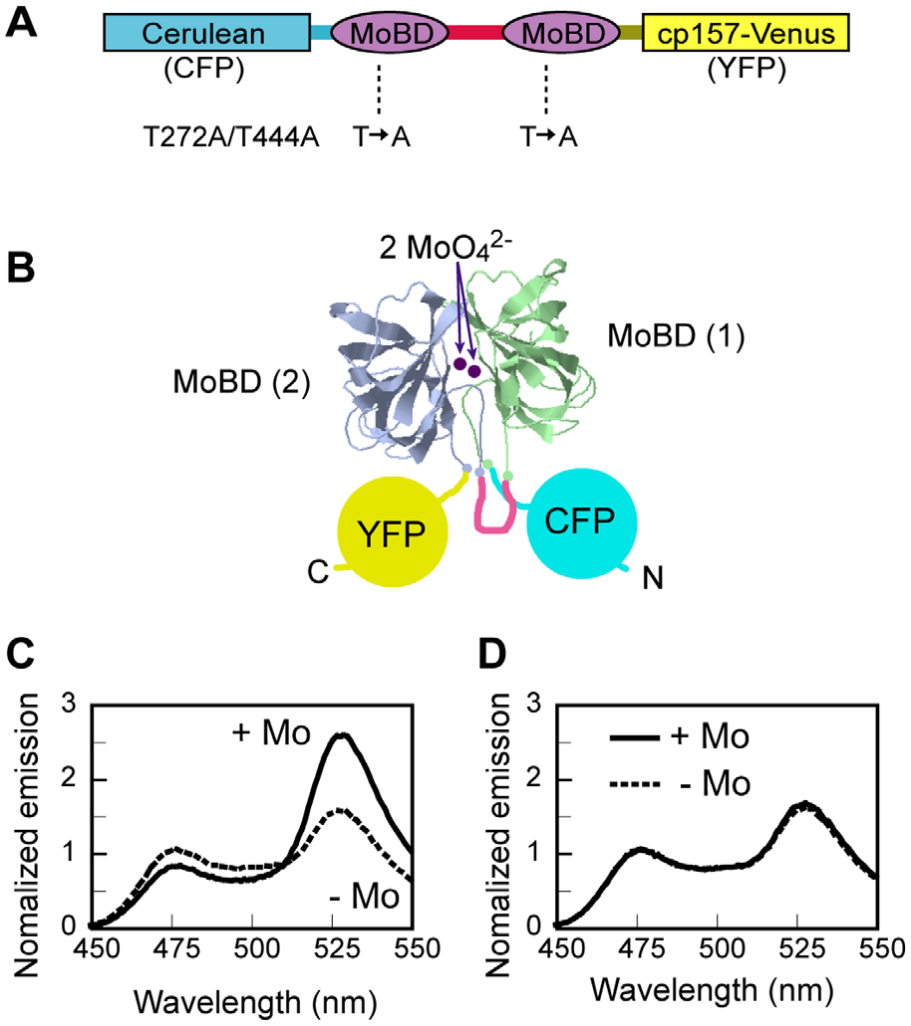
FRET-based genetically encoded nanosensor for molybdate. A , Primary structure of MolyProbe. CFP (Cerulean), two molybdate binding domains (MoBD) and YFP (cp157-Venus) are connected by optimized peptide linkers. MoBDs are from *E.coli* ModE factor. T272A/T444A is a loss-of-function mutant. **B**, Schematic representation of molybdate binding between two MoBDs, which increase FRET efficiency. **C**, Spectral property of MolyProbe *in vitro*. The emission spectrum of recombinant protein (20 nM) was measured at λ_Ex_ 430 nm (λ_max_ for CFP), with or without 10 µM molybdate. **D**, Emission spectral property of the T272A/T444A double mutant.

A genetically-encoded FRET nanosensor for molybdate was constructed by CFP-variant Cerulean [23], YFP-variant cp157-Venus [24] and a molybdate binding domain (MoBD) (Figure 1A). MoBD consists of the C-terminal end (122–262 aa) of the *E.coli* transcription factor ModE, which still possesses molybdate-binding-dependent homo- dimerization capability, but lacks DNA binding [18], [19]. In order to simulate MoBD dimerization in a single molecule, a pair of MoBDs was linked in tandem by way of a peptide linker, resulting in close positioning of the N- and C-termini in the presence of molybdate. This conformation was expected due to the proximity of residue Ser-122 of one protomer and Cys-262 of another, as seen in the crystal structure of the ‘complete’ ModE homo dimer (PDB#1O7L) [19]. CFP, a FRET-donor, and YFP, a FRET-acceptor, were then fused to the N- and C-termini of the linked MoBD pair respectively. The intra-molecular assembly of the two MoBDs induced by molybdate was expected to reduce the distance between CFP and YFP, and/or narrow the solid angle between the two chromophores, thus increasing FRET efficiency (Figure 1B).

The hypothesized resulting functionality was illustrated by excitation at 430 nm, at which the recombinant protein emitted a fluorescence spectrum with two peaks corresponding to CFP (475 nm) and YFP (530 nm). The emission intensity ratio (R_F530:F475_) changed in the presence or absence of molybdate. However, the dynamic R_F530:F475_ range of the initial prototype was not broad enough to permit quantitative analysis of fluorescence *in vivo* (data not shown). We therefore improved the prototype sensor by optimizing the peptide linkers between MoBD, CFP and YFP, and introducing a circular permutation in YFP. The modified sensors were expressed in *E.coli*, purified, their R_F530:F475_ dynamic ranges tested, and the best one was selected and designated MolyProbe (accession no. AB673363). The R_F530:F475_ of MolyProbe lacking molybdate was 1.58 due to basal FRET. The R_F530:F475_ showed a dramatic (109%) increase to 3.31 by addition of molybdate at 10 µM (Figure 1C). This R_F530:F475_ change was completely abolished by a T272A/T444A double mutation introduced in the molybdate binding site of both MoBDs. The R_F530:F475_ of the T272A/T444A mutant was always 1.6, almost the same as the basal rate of the wild type MolyProbe (Figure 1D).

### Biochemical Evaluation of MolyProbe in vitro

**Figure 2:**
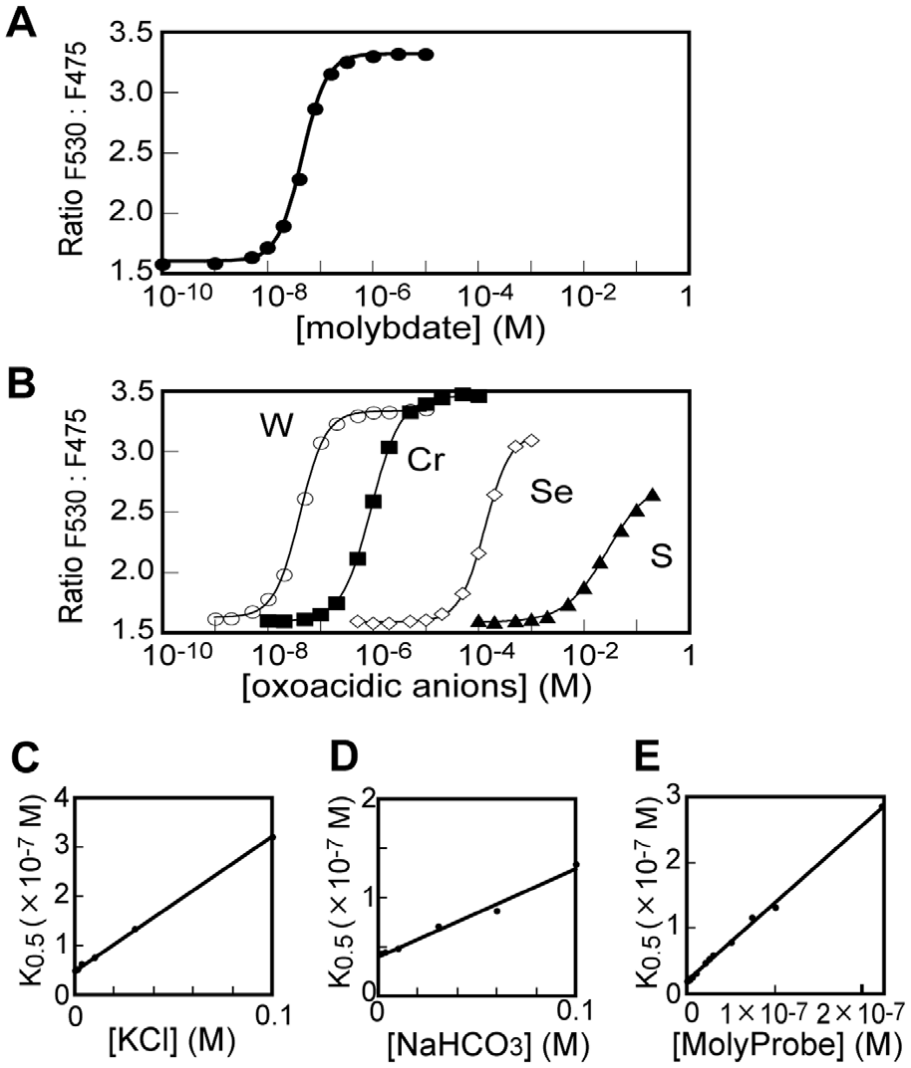
Sensitivity and specificity of MolyProbe. A , Titration curve of MolyProbe to molybdate. Emission spectrum was measured as described in Figure 1. Emission intensity ratio (F530: F475) was calculated and plotted against molybdate concentration. The plot was fitted by the Hill equation. **B**, Titration curves for similar oxyanions. **C**, Inhibitory effect of chloride. Titration curves for molybdate were determined in the presence of 1–100 mM KCl. Apparent *K*_0.5_ values for molybdate were calculated and plotted against concentrations of potassium chloride. **D**, Inhibitory effect of bicarbonate. **E**, Inhibitory effect of MolyProbe. Titration curves were determined at a concentration of 0.5–200 nM MolyProbe. Average data were obtained by triplicate assays. The SDs were small (<2% for A, B and <5% for C–E).

A recombinant MolyProbe protein was purified (>99%) and analyzed for biochemical properties including molybdate and inhibitor specificity and affinity, reversibility of *in vitro* ligand binding and time-resolution. MolyProbe was titrated by molybdate and other oxyanions similar to molybdate, and the apparent dissociation constant was calculated from the data by the Hill equation. Since the MolyProbe protein possesses two MoBD moieties interacting with each other, the following equation was adopted:
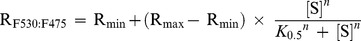
where [S] is the substrate concentration; R_min_, minimum of R_F530:F475_; R_max_, maximum of R_F530:F475_; *K*_0.5_, an apparent dissociation constant; n, Hill coefficient. Under our standard assay conditions, the R_F530:F475_ of MolyProbe (20 nM) ranged from 1.58 to 3.31 by adding 10^−8^∼10^−7^ M molybdate (MoO_4_^2−^), giving an apparent *K*_0.5_ for molybdate of 4.7×10^−8^ M and a Hill coefficient of 1.9 (Figure 2A). Other oxyanions which share a sp3-hybridized tetrahedral structure with molybdate also increased R_F530:F475_ (Figure 2B), obeying the apparent dissociation constants as follows: a 4.1×10^−8^ M *K*_0.5_ for tungstate (WO_4_^2−^); a 9.2×10^−7^ M apparent *K*_0.5_ for chromate (CrO_4_^2−^); a 1.4×10^−4^ M for selenate (SeO_4_^2−^); and a 2.7×10^−2^ M for sulfate (SO_4_^2−^). The affinity for tungstate was similar to that for molybdate, while the affinities for other oxyanions were one to six orders of magnitude lower than that for molybdate. Biologically abundant anions such as chloride, bicarbonate, nitrate, acetate, glutamate and phosphate, did not increase R_F530:F475_ independently.

Chloride and bicarbonate resulted in a shift in the titration curve for molybdate to a higher concentration, indicating a competitive inhibitory effect. We thus examined values of apparent *K*_0.5_ for molybdate in the presence of 1–100 mM chloride or bicarbonate (Figure 2C, D). The curve was also horizontally migrated by varying the concentration of the MolyProbe itself, because sensor molecules compete with each other for ligand binding. We therefore obtained the apparent *K*_0.5_ at a concentration of 0.5–200 nM MolyProbe (Figure 2E). The data were analyzed by way of the following Equation:
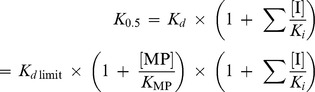
where *K*_0.5_ is an apparent dissociation constant observed; *K*_d_, a calculated dissociation constant without inhibitors; [I], concentration of inhibitory ion; and *K*_i_, an inhibitor constant; *K*_d limit_, a calculated dissociation constant with a dilution limit MolyProbe and without inhibitors; [MP], concentration of MolyProbe and *K*_MP_. a competition constant of MolyProbe itself. The *K*_i_ for chloride and bicarbonate were 1.4×10^−2^ M and 3.3×10^−2^ M, respectively. The *K*_d limit_ and *K*_MP_ were calculated as 1.7×10^−8^ M and 1.6×10^−8^ M, respectively.

In order to determine the time response of MolyProbe, pre-steady-state output changes immediately after molybdate addition or dilution were analyzed. Because the change rate was too fast to detect two channel fluorescence providing R_F530:F475_, we only monitored the time-course of YFP fluorescence (F_530_). The F_530_ rapidly increased by addition of 100 nM molybdate to the buffer, and equilibrated with a half-time of 4.8 sec. In contrast, the F_530_ decreased relatively slowly with a decreasing molybdate concentration from 60 to 12 nM, and equilibrated in a half-time of 25.3 sec, indicating molybdate release was five-fold slower than binding.

### Molybdate Sensing in Living Mammalian Cells

**Figure 3:**
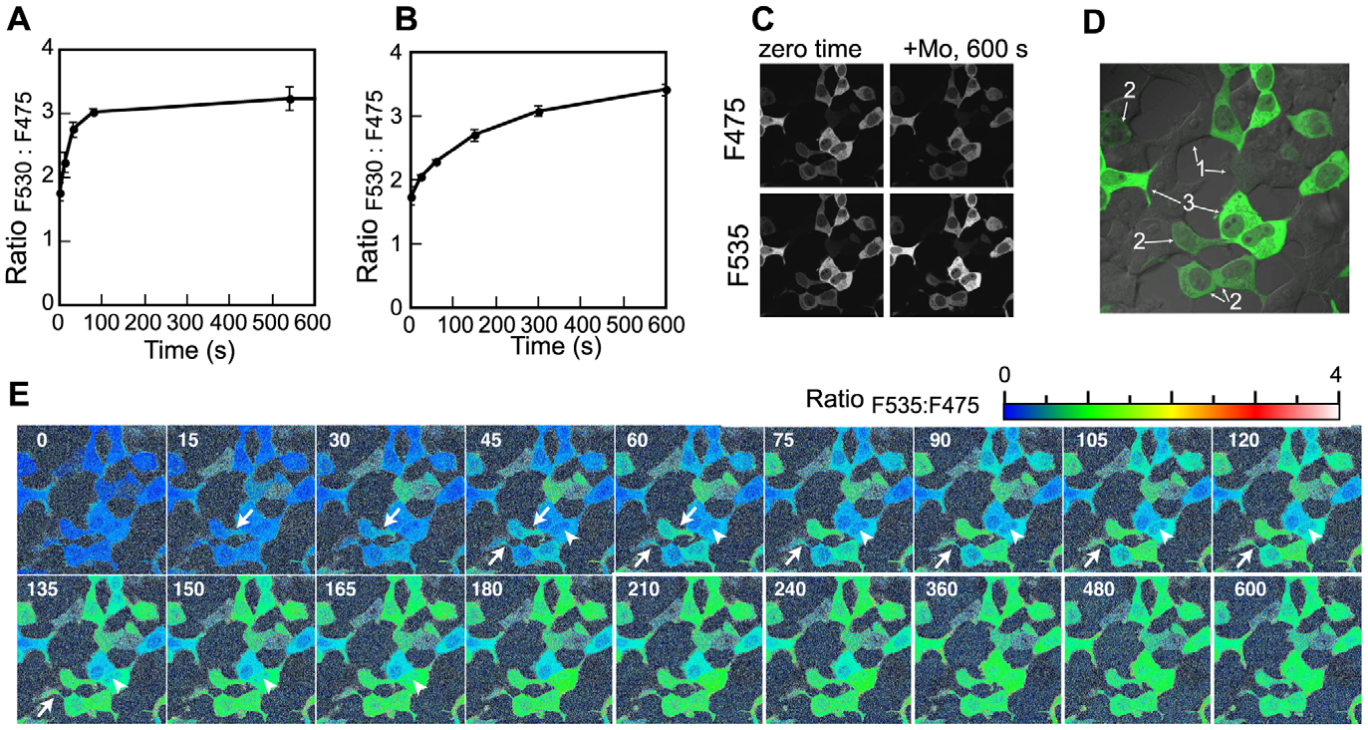
Real time imaging of molybdate level in living animal cells. A , Time course of R_F530:F475_ in bulk HEK-293T cells transfected with MolyProbe after exposure to 1 mM molybdate at 37°C. **B**, Time course of R_F530:F475_ in the bulk cells treated with 1 mM molybdate at 22°C. Averages and SDs from triplicate samples are shown. **C**, Confocal CFP(F475) and YFP(F535) images of the cells before and after treatment with 1 mM molybdate. **D**, Variation in expression levels of MolyProbe. A density image of MolyProbe (green) calculated from a pair of CFP and YFP image was merged with a transmission image (grey). Low-level expression cells (1), middle-level cells (2) and high-level cells (3) showed a different time course of the ratio change (Figure E). **E**, A series of ratio images of the cells accumulating molybdate. Ratio (F535:F475) images were calculated from pairs of CFP and YFP images taken every 15 sec, and the ratios are presented in pseudo-color. Intracellular molybdate increased by addition of 1 mM molybdate to the medium. Velocity of the molybdate increment in pseudopod was fast compared to the cell body (arrow), whereas nuclear space was slow (arrowhead).

Having developed the MolyProbe, we sought to investigate its ability to detect molybdate in living mammalian cells. HEK-293T cells were transiently transfected with MolyProbe and subjected to a bulk assay. When a suspension of the intact cells was excited at 430 nm, the characteristic MolyProbe fluorescence spectrum was observed, corresponding to CFP and YFP (data not shown). The basal R_F530:F475_ was 1.8±0.05, rapidly increasing upon addition of excess molybdate (1 mM) to the medium, reaching a saturation level of 3 within 2 minutes at 37°C (Figure 3A) and 10 minutes at 22°C (Figure 3B), respectively.

We then observed real-time dynamics of molybdate at the single cell level by FRET ratio imaging using a laser-scanning confocal microscope (LSCM) at 22°C. The MolyProbe was found to be predominantly distributed in the cytoplasm of the transfected cells, and, to a lesser degree, in the nucleus (Figure 3C, D). By addition of 1 mM molybdate into the culture medium, the fluorescence intensity of CFP in the cells decreased in a time-dependent manner, whereas YFP fluorescence intensity increased (Figure 3C). Hence the emission intensity ratio (R_F535:F475_) acquired by LSCM was shown to sequentially increase (Figure 3E). At the single cell level, the rate of the R_F535:F475_ change also differed according to subcellular localization; for instance, the R_F535:F475_ change observed inside pseudopods stretching from cells was found to be relatively faster than that inside the cell body (Figure 3E, arrow). The nuclear R_F535:F475_ change rate was relatively slow compared to that observed within the cytoplasm (Figure 3E, arrowhead). Note that the MolyProbe protein, detected as a 90 kD band on the immunoblot, remained constant during the live cell assays, indicating that no partial degradation had taken place (data not shown).

### Characterization of Molybdate Uptake in the Culture Cell

**Figure 4:**
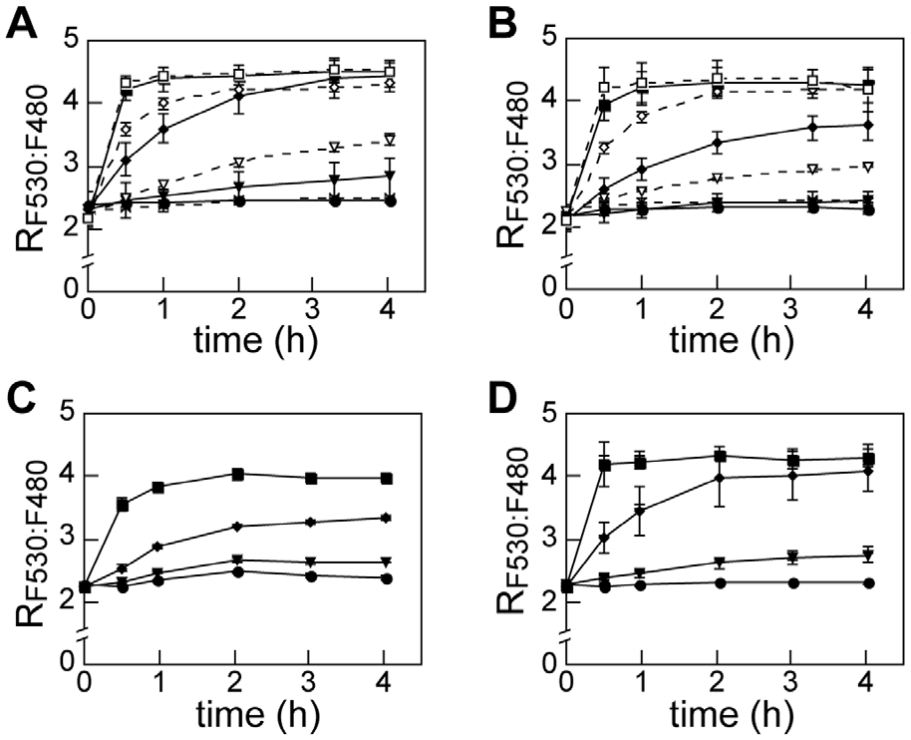
Molybdate accumulation *in vivo*. A , Time course of R_F530:F480_ in living animal cells at 37°C responded to different doses of molybdate. HEK-293T transfected with MolyProbe was treated by molybdate at the concentrations indicated. Fluorescence of the cell was measured at 0, 0.5, 1, 2, 3, 4 hr, and R_F530:F480_ calculated. **B**, Effect of medium exchange on the time course. The cell medium was replaced by fresh D-MEM/F-12/FBS medium prior to the assay. **C**, Time course of R_F530:F480_ in the presence of 10 mM oxalate. **D**, Time course of R_F530:F480_ supplemented with sulfate (1 mM). Concentrations of molybdate in the working medium are as follows: 0 µM (closed circle), 0.1 µM (cross), 0.3 µM (closed triangle), 1 µM (open triangle), 3 µM (closed diamond), 10 µM (open diamond), 30 µM (closed square), 100 µM (open square). Averages and SDs from triplicate samples are shown. n = 3.

Finally we roughly characterized molybdate uptake of the animal cell by carrying out a series of time-course experiments *in vivo*. MolyProbe expressing cells were treated with molybdate at different doses (0.1–100 µM), and then the FRET-ratios (R_F530:F480_) were measured sequentially. The ratio increased in both a time- and molybdate-dose (from 0.3 µM to 30 µM)- dependent manner (Figure 4A). Interestingly, refreshment of the culture medium prior to the time-course assay slightly weakened the observed ratio increase (Figure 4B). We then tested inhibitory effects of components that consist the DMEM/F-12 medium. One of the components, pyruvate (0.5 mM) exhibited similar effect on the ratio change (data not shown). Because pyruvate have carboxylate group, we next investigated the inhibitory effects of other carboxylate anions. Among inhibitors screened, oxalate (10 mM) strongly lower-shifted the R_F530:F480_ curves (Figure 4C). Sulfate (1 mM), a component of the DMEM/F-12 medium (0.5 mM), showed little effect on the molybdate uptake (Figure 4D), although it was reported as a competitive inhibitor of molybdate transport in some cell types because of its structural similarity [29], [30]. Note that increase of total molybdenum content in cell factions at typical points of the time-course experiment were confirmed by ICP-MS analysis or MolyProbe *in vitro* assay (data not shown). We also confirmed both absence of tungsten (<10 nM) in the cell and the inhibitory-effect of oxalate (10 mM) for molybdenum intake by measuring molybdenum or tungsten in prepared cell pellets with ICP-MS (data not shown).

**Figure 5:**
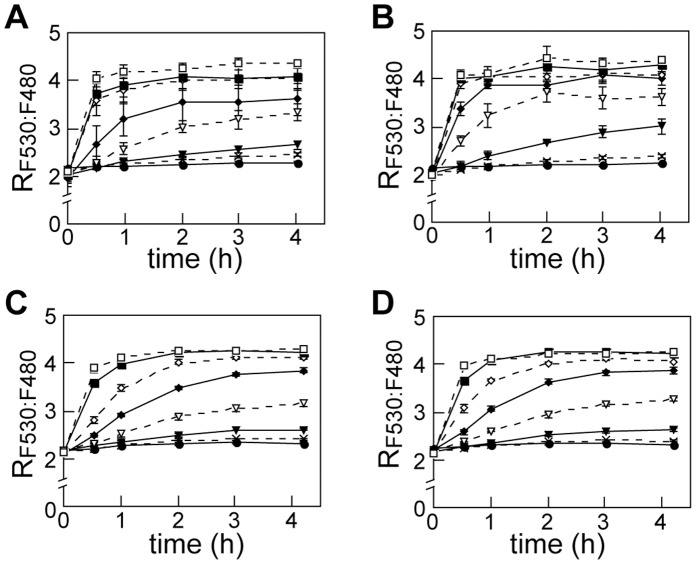
Effect of over-expression and knockdown of *Hs*MoT2/MFSD5 in molybdate uptake rate. A , Time course of R_F530:F480_ in control cells (for Panel B). HEK-293T was co-transfected with MolyProbe and mock vector by the polyethyleneimine method. Fluorescence was measured after addition of molybdate, and R_F530:F480_ calculated. **B**, Time course of *Hs*MoT2/MFSD5 over-expressing cells. mRNA level of *Hs*MoT2/MFSD5 was about sixty-fold compared to the control cell. **C**, Time course of R_F530:F480_ in control cells (for Panel D) transfected with MolyProbe by X-tremeGENE siRNA reagent. **D**, Time course of R_F530:F480_ in *Hs*MoT2/MFSD5 knockdown cells. mRNA level of *Hs*MoT2/MFSD5 was 11–35% compared to the control cell. Concentrations of molybdate in working medium are follows: 0 µM (closed circle), 0.1 µM (cross), 0.3 µM (closed triangle), 1 µM (open triangle), 3 µM (closed diamond), 10 µM (open diamond), 30 µM (closed square), 100 µM (open square). Averages and SDs from triplicate samples are shown. n = 3.

Recently, *Hs*MoT2/MFSD5, a human homolog of an algal molybdate transporter *Cr*MoT2, had been reported to exhibit molybdate uptake activity in a yeast over-expression system [31]. As *Hs*MoT2 mRNA was detected in HEK-293T (5.9±1.5×10^5^ copy/mg total RNA), we assessed whether *Hs*MoT2 was responsible for molybdate uptake by the cell. A sixty-fold over-expression of *Hs*MoT2 upper-shifted the R_F530:F480_ curves, with a molybdate input in the 0.3 mM to 10 mM range (Figure 5A, B). Knockdown of endogenous *Hs*MoT2 by small interfering RNA resulted in a decrease in mRNA (11–35% of control). However, this was not reflected in a lower-shift of the R_F530:F480_ curves at any dose of molybdate (Figure 5C, D).

## Discussion

In this study, we developed a genetically-encoded nanosensor based on CFP-YFP hetero-FRET to detect trace molybdate both *in vitro* and *in vivo*. All the components of the molybdate nanosensor were assembled in a single polypeptide, because homo-dimerization of MoBD in the same molecule was expected to result in steady pairing of CFP and YFP. A single type FRET sensor such as MolyProbe must be theoretically superior to a split-type nanosensor composed of a pair of separate CFP::MoBD and YFP::MoBD molecules. The probability of the CFP-YFP pairing in the single type nanosensor is nearly 100%, while that of the split-type nanosensor is expected to be less than 50%. The remaining pairing should consist of CFP-CFP and/or YFP-YFP pairs [32]. Moreover, MolyProbe unexpectedly improved ligand affinity. More specifically, the *K*_d_limit_ of MolyProbe for molybdate was found to be 17 nM; that is one-order lower than that of the native ModE protein (0.8 µM [21]) and of the truncated MoBD protein, (0.5 µM [18]). Restriction of free diffusion of the two MoBDs by an appropriate physical linkage may increase the probability of encountering two molybdate-binding MoBDs, resulting in higher affinity. Note that, another prototype sensor including a single MoBD, with a primary structure of CFP::MoBD::YFP (#S1-2B) was found to have a sub-micro molar *K*_d_ for molybdate, comparable to that of the native ModE (data not shown). The affinity is sufficient for molybdate measurement *in vivo*. Concentrations of total molybdenum in animal tissues/cells have been reported, with values ranging from about 50 to 1000 ppb (0.5 to 10 µM) [33]–[36]. The values are one- to two-order higher than those of body fluids (serum, urine, milk) ranging from about 1 to 50 ppb (10 to 500 nM) [37]–[40]. Part of the molybdenum in cells may exist as molybdate, although the molar ratio of molybdate against total molybdenum have not known yet. Moreover, with regard to substrate specificity, MolyProbe could be used to detect molybdate *in vivo*. Although MolyProbe is activated both by molybdate and sp3-hybridyzed oxyanions, the influences of the other oxyanions may be small or negligible *in vivo*. In fact, the apparent *K*_0.5_ for tungstate (4.1×10^−8^ M) was comparable to that observed for molybdate (4.7×10^−8^ M). However, abundance of tungsten in the biological environment is two orders of magnitude lower than that of molybdenum [1]. On the other hand, among the remaining oxyanions, sulfate alone shows strong abundance in the cytosol, with a concentration of 1×10^−3^ M [41]. Because the apparent *K*_0.5_ for sulfate is 2.2×10^−2^ M, there is a chance that, albeit to a small degree, it may interfere with molybdate *in vivo*. The responsiveness of MolyProbe is sufficiently fast to monitor dynamic change of molybdate in living cells at the sub-minute level. In fact, sub-cellular distributions of molybdate were transiently observed in the real-time imaging analysis. This means that the responsiveness of MolyProbe is faster than the diffusion rate of molybdate in cytosol.

Time-course analyses of MolyProbe in living HEK-293T cells pointed to the putative presence of membrane transport(er)s for regulation of molybdate concentration inside cells. Even though excess molybdate (1 mM) was added into the medium, the intracellular MolyProbe response was observed to be in the order of minutes. The speed of the response was also shown to be temperature dependent (37°C, Figure 3A vs 22°C, Figure 3B). The slow, temperature-dependent increase in molybdate suggests that molybdate is not imported by diffusion, but by membrane transport. Moreover, a series of dynamic analyses allowed determination of the characteristics of a molybdate transporter in the HEK-293T cells; it recognizes sub-micro to micro-molar range (0.3 µM to 30 µM) substrate, and is disrupted by some components of fresh medium or oxalate (10 mM).

The oxalate-sensitive molybdate transporter in the culture cell is a novel type one different from known molybdate transporters in animal cells. Because mammalian molybdate transporters are yet to be fully identified, further investigations are required in order to better understand the uptake of molybdate in the cells. Mammals lack any homologues of the bacterial ModABC-type transporter [2], [5]. Strict sulfate transporter should also be excluded as a candidate, although molybdate and sulfate share a tetrahedral structure, because the uptake of molybdate observed in this study is resistant to sulfate. Recently, two different molybdate transporter families, green plant MOT1/MOT2/MoT1 and algal MoT2, have been identified [42]–[44], [31]. Among them, the MoT2 family contains mammalian members, human homolog *Hs*MoT2/MFSD5 for example; which is thought to be responsible for molybdate uptake in mammalian cells. We confirmed the ability of *Hs*MoT2/MFSD5 to accelerate molybdate intake by over-expression of the molecule. Our knockdown experiment, however, indicates that *Hs*MoT2/MFSD5 rarely contributes to oxalate-sensitive molybdate uptake in the HEK-293 T cells, although detectable amounts of *Hs*MoT2/MFSD5 mRNA exist (about 10 copy/cell).

The MolyProbe nanosensor was shown to be suitable for detecting trace level molybdate both *in vitro* and *in vivo*. More detailed analyses of molybdate dynamics may reveal more about the actual concentration, flux, regulation and specific membrane transport of molybdate in mammalian cells.
